# Pennsylvania Hospital

**Published:** 1842-01-15

**Authors:** 


PENNSYLVANIA HOSPITAL—*Surgical Wards—Service of* DR. E. PEACE.

1. *Fracture of the humerus with separation of the condyles.*—Discharged, January 1st, a case of transverse fracture of the humerus near the condyles, with a longitudinal fracture separating the inner condyle from the shaft of the bone, in a child, æt. 5, and attended with considerable inflammation of the elbow-joint. The inflammation was subdued with leeches and the lead-water lotion. The fracture was treated with a jointed splint, fixed, in the first instance, at a right angle, and applied to the inner side of the limb. Three small splints were adjusted severally to the three other faces of the humerus. After the first week, the angle of the inner splint was gradually increased, so as to extend the limb. Passive motion of the forearm was begun at the end of the second week, by which time union appeared to have become quite firm. The small splints were removed in three weeks, and the jointed splint in thirty days. Some rigidity of the joint remained. This was gradually overcome so far as to enable the patient to extend the limb almost completely, but not to flex it further than to an angle of about forty-five degrees. No deformity was apparent. The child remained in the ward thirty days after the splints were taken off, on account of the imperfect motion of the joint. He was discharged at last without having recovered the power of entirely flexing his arm, although he apppeared to be slowly regaining this also. E. H.

